# Developing a rehabilitation intervention difficulty index: A mixed-methods study using NASA-TLX and Borg RPE in a tertiary clinical setting

**DOI:** 10.1371/journal.pone.0340770

**Published:** 2026-01-12

**Authors:** Yazeed Temraz, Theeb Al Salem, Yousef Al Nufaie, Abdullah Aaltaza, Muhanna Almandoor, Turkey Al-Subaie, Sama Alshalawi, Saud Alsaadoon, Abdulrahman Al Hussein, Mohammed Aldakhil

**Affiliations:** 1 Rehabilitation Department, Ministry of the National Guard-Health Affairs, Riyadh, Saudi Arabia; 2 King Abdullah International Medical Research Center (KAIMRC), Riyadh, Saudi Arabia; University of Maryland at College Park: University of Maryland, UNITED STATES OF AMERICA

## Abstract

**Background:**

Rehabilitation difficulty varies substantially across clinical areas and intervention types, yet few brief, standardised measures exist to quantify difficulty from the therapist’s perspective. Understanding rehabilitation difficulty is important for workforce planning, resource allocation, and identifying contexts where therapist support is most needed.

**Objective:**

To develop and preliminarily evaluate the Rehabilitation Intervention Difficulty Index (RIDI), a mixed-methods measure combining cognitive/affective and physiological dimensions of rehabilitation difficulty, and to identify contextual factors associated with high difficulty.

**Methods:**

*Quantitative component:* 441 rehabilitation sessions from 28 therapists across 12 clinical areas were assessed using the NASA Task Load Index (NASA-TLX; four items: mental demand, temporal demand, effort, frustration) and Borg Rating of Perceived Exertion (RPE). The RIDI was constructed as the mean of the NASA composite score and normalised Borg RPE (0–10 scale). Psychometric properties were evaluated using Cronbach’s alpha and exploratory factor analysis. Descriptive statistics, one-way ANOVA, Pearson correlations, and intervention-level summaries were performed.

*Qualitative component:* Semi-structured interviews and focus groups with 20 therapists (10 individual interviews, 10 focus-group participants; 11 physiotherapists, 9 occupational therapists) were conducted across selected clinical areas in the same hospital. Thematic analysis identified key sources of rehabilitation difficulty. Qualitative and quantitative findings were integrated through joint display analysis.

**Results:**

*Quantitative findings:* RIDI showed good internal consistency for the NASA items (α = 0.80, 95% CI [0.77, 0.83]) and acceptable consistency for the five-item set (α = 0.75, 95% CI [0.72, 0.78]), with a unidimensional factor structure explaining 52% of the variance. RIDI scores differed significantly across clinical areas (F = 9.18, p < 0.001, η² = 0.19), with higher scores in neurorehabilitation and acute neuro settings (both 5.80) and lower scores in outpatient physical therapy (3.63). Among frequently used interventions (n ≥ 5), transfer training and splinting had the highest RIDI scores (6.78 and 6.71), while passive range of motion had the lowest (2.68). RIDI was moderately correlated with session duration (r = 0.28, p < 0.001) but not with therapist experience (r = 0.01, p = 0.78).*Qualitative findings:* Six themes described sources of difficulty: time demands (78 coded segments, 18 participants), cognitive demands (72, 18), physical demands (65, 17), patient-related factors (57, 16), environmental constraints (44, 15), and coping strategies (33, 14). No substantially new themes were identified after approximately 70% of the data, suggesting thematic saturation.*Mixed-methods integration:* High RIDI scores in neuro and acute inpatient areas converged with qualitative descriptions of heavy time, cognitive, and physical demands. Coping strategies were discussed much less often in high-RIDI areas (8 segments) than in lower-RIDI areas (23 segments), suggesting that in the most demanding contexts therapists may have fewer opportunities to apply deliberate coping strategies.

**Conclusions:**

RIDI showed acceptable reliability and preliminary evidence of construct validity as a therapist-reported measure of rehabilitation difficulty that captures both cognitive/affective and physiological aspects of workload. Variation in RIDI scores across clinical areas and interventions was consistent with meaningful differences in perceived difficulty. The under-representation of coping strategies in high-RIDI contexts suggests that high rehabilitation difficulty may require structural and organisational responses—such as staffing, workflow redesign, and environmental modifications—rather than relying primarily on individual coping. RIDI may provide a practical tool for identifying high-demand areas, informing workforce planning, and targeting support where it is most needed.

## Introduction

Rehabilitation is a labour-intensive part of healthcare, delivered through complex, multi-component interventions that place substantial mental, physical and decision-making demands on therapists [1–3]. Physical and occupational therapists often manage medically complex patients and competing clinical priorities in fast-paced environments and under considerable time pressure [4,5]. Intervention complexity and workload vary across settings and intervention types, from brief standardised procedures to multi-step, high-complexity interventions in acute and intensive care [3,6,7]. Existing workload systems in rehabilitation are heterogeneous and usually focus on time or visit counts rather than perceived intervention difficulty, offering limited support for evidence-based workforce planning and allocation of support [2,4–6,8,9].

Workload assessment is central to healthcare quality because workload metrics can inform monitoring of patient safety, clinician performance and staffing decisions [10–12]. High workload has been linked to low job satisfaction, burnout, staff turnover and, in nursing, higher risks of adverse patient outcomes such as medication errors, infections and mortality [10,13–15]. Conversely, a more balanced distribution of workload, supported by adequate resources, is associated with better staff well-being and more efficient use of services, consistent with Job Demands–Resources (JD-R) evidence that sufficient resources buffer the impact of high demands on burnout and performance [11,13–15]. Process-improvement work in rehabilitation further shows that meaningful, context-specific productivity metrics are crucial for engaging therapists and supporting efficiency initiatives [16].

In Saudi Arabia, rehabilitation services have expanded as part of the Vision 2030 health reforms, yet international and local data still show high rates of work-related musculoskeletal disorders and burnout among rehabilitation therapists, raising concerns about workforce sustainability [17–21]. Despite this, there is no standard, brief way to capture the difficulty of rehabilitation interventions from the therapist’s perspective, leaving administrators with limited data to guide staffing, protect therapist health and plan service delivery, even as workload tools are increasingly recommended for workforce planning and quality improvement in primary care and hospital services [8,10,12].

Several approaches to measuring workload have been used in healthcare. Objective measures such as patient–staff ratios, procedure counts and direct care time provide clear numerical estimates but may not reflect how difficult the work feels to clinicians [10]. Subjective ratings of workload capture clinicians’ experiences but can vary between individuals, while physiological indicators such as heart-rate-based metrics can signal stress responses yet are difficult to apply in routine practice [10]. In other disciplines, composite indices—including nursing workload scores, primary-care workload instruments and rehabilitation case-complexity models—have been used to support staff planning, standardise assessments and guide allocation of resources [7,10,12,22]. Rehabilitation currently lacks an equivalent, validated composite index focused specifically on the difficulty of treatment sessions from the therapist’s perspective.

Two established tools are particularly relevant for rehabilitation practice. The NASA Task Load Index (NASA-TLX) is a multidimensional rating scale of subjective workload with six subscales: mental demand, physical demand, temporal demand, performance, effort and frustration [23]. It has been widely applied and validated in healthcare settings, including ICU nursing, patient-monitoring tasks and surgical procedures [24–29]. The Borg Rating of Perceived Exertion (RPE) is a self-report scale widely used in exercise physiology, cardiac rehabilitation and other rehabilitation contexts to quantify perceived physical effort and to guide exercise intensity and training loads [30–36]. Together, NASA-TLX and Borg RPE capture cognitive/affective and physiological aspects of work, but neither was designed to summarise the overall difficulty of rehabilitation interventions at the session level.

**Fig 1:**
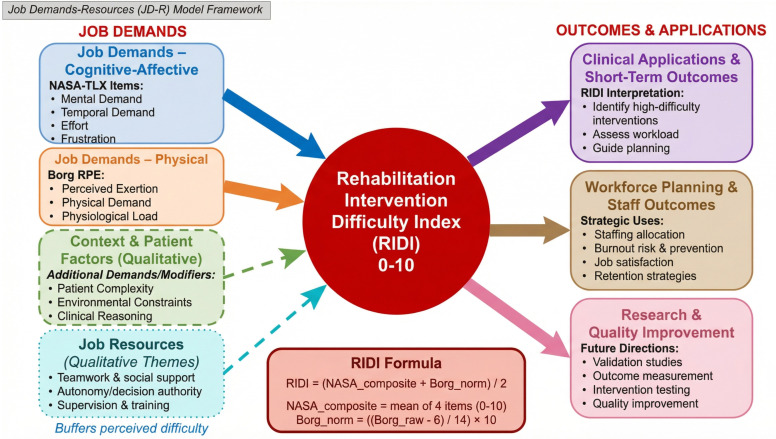
Conceptual framework for the Rehabilitation Intervention Difficulty Index (RIDI) within the Job Demands–Resources (JD-R) model. RIDI (0–10) summarises session-level intervention difficulty by combining cognitive–affective demands from selected NASA-TLX items and physical demands from the Borg Rating of Perceived Exertion, using the formula shown. Context and patient factors, together with job resources such as teamwork, autonomy and supervision, modify perceived difficulty in line with the JD-R model. The resulting RIDI scores can inform clinical decision-making, workforce planning and future research and quality-improvement initiatives.

Within the JD-R framework, rehabilitation intervention difficulty can be viewed as the level of job demands experienced during a single treatment session, whereas coping strategies, teamwork, autonomy and organisational support represent job resources that may buffer these demands [13–15]. The Rehabilitation Intervention Difficulty Index (RIDI) focuses on quantifying session-level demands (cognitive–affective and physical load), while the qualitative component captures contextual demands and resources that shape how difficulty is experienced. Fig 1 presents a conceptual framework based on the JD-R model, linking NASA-TLX and Borg RPE components, contextual factors and potential clinical and organisational applications of RIDI.

On this basis, the Rehabilitation Intervention Difficulty Index (RIDI) was developed, combining selected NASA-TLX items (mental demand, temporal demand, effort, frustration) with the Borg RPE to create a single 0–10 score for perceived intervention difficulty. A mixed-methods design was adopted because quantitative measures alone rarely explain how or why particular interventions are experienced as more or less demanding in specific clinical contexts [37–39]. Qualitative interviews and a focus group provide insight into how therapists experience and manage difficult sessions and help interpret patterns in RIDI scores.

The aim of this mixed-methods study was to develop and preliminarily evaluate the Rehabilitation Intervention Difficulty Index in a tertiary rehabilitation service. The specific objectives were to: (1) construct a numerical index of intervention difficulty using NASA-TLX and Borg RPE; (2) examine its preliminary psychometric properties (internal consistency and factor structure); (3) describe variation in RIDI scores across clinical areas, intervention types and therapist characteristics; and (4) identify key sources of rehabilitation difficulty through qualitative interviews and a focus group, and integrate these findings with the quantitative patterns.

## Methods

### Study design

A convergent parallel mixed-methods design was used to develop and preliminarily evaluate the Rehabilitation Intervention Difficulty Index (RIDI). The quantitative component was a cross-sectional assessment of 441 rehabilitation sessions across 12 clinical areas; the qualitative component comprised semi-structured interviews and one focus group with 20 therapists from the same areas. Data were collected during routine practice over four months (April–July 2025). Reporting follows STROBE for observational studies and COREQ for qualitative research; completed checklists are provided in the supplementary materials.

### Quantitative component

#### Study setting and participants.

The study was conducted at the Ministry of National Guard Health Affairs Hospital in Riyadh, Saudi Arabia, a 1,500-bed tertiary teaching and referral hospital. The rehabilitation department provides multidisciplinary physiotherapy and occupational therapy services in 12 clinical areas, including intensive care, acute neurology, burn care, internal medicine, orthopaedic rehabilitation, neurorehabilitation outpatients and other specialised units. Twenty-eight physiotherapists and occupational therapists completed RIDI assessments for 441 consecutive sessions, sampled to reflect the range of clinical areas and intervention types routinely delivered.

#### Rehabilitation interventions (usual care).

Across the 12 areas, rehabilitation followed usual-care rather than standardised research protocols. Physiotherapy typically included mobility and gait training, transfers, strengthening and conditioning, balance training, respiratory physiotherapy and pain-management techniques. Occupational therapy focused on activities of daily living (ADL) retraining, upper-limb and fine motor tasks, cognitive and perceptual rehabilitation, environmental modification and education in the use of adaptive equipment. Each RIDI score therefore reflected the perceived difficulty of a routine treatment session in its real clinical context.

#### Sample size considerations.

The quantitative target was ≥ 400 sessions, based on feasibility and common recommendations of 5–10 observations per item for exploratory factor analysis and internal consistency testing [40,41]. With five RIDI component items and 441 sessions (≈88 observations per item), the sample exceeded these guidelines and was adequate for correlation and variance analyses typical of validation studies of subjective workload measures.

#### Data cleaning and preparation.

Data were extracted from therapist-completed electronic forms. No missing or out-of-range values were observed for key variables (NASA-TLX items, Borg RPE, session duration, clinical area, intervention label and therapist characteristics). Inconsistent “years of experience” entries for two therapists were resolved by retaining the most frequent value. Free-text intervention labels were cleaned and harmonised (e.g., spelling corrections and merging obvious variants), reducing 135 raw labels to 122 standardised categories; details presented in S1 Table.

### Measures: NASA-TLX, Borg RPE, and RIDI construction

#### NASA-TLX and Borg RPE.

After each session, therapists rated four NASA-TLX items (mental demand, temporal demand, effort, frustration) on 0–10 scales; their mean formed a NASA composite score (0–10). Physical effort was rated using the Borg Rating of Perceived Exertion (RPE) on the 6–20 scale. NASA-TLX and Borg RPE were used as published and appropriately cited. The manuscript does not reproduce proprietary scoring forms or copyrighted graphical materials; therefore, no additional permissions were required for use of these instruments in this study. Standard wording and scoring recommended by the original authors were used. Borg scores were normalised to a 0–10 range using:


Borg_norm = ((Borg_raw − 6)/ 14) × 10

So that 6 corresponds to 0 and 20–10.

#### Rehabilitation intervention difficulty index (RIDI).

RIDI was designed as a single indicator of perceived intervention difficulty that combines cognitive–affective load and physical exertion. For each session:


RIDI = (NASA_composite + Borg_norm)/ 2

yielding a 0–10 scale, where higher scores indicate greater perceived difficulty. The RIDI development process involved expert consultation with senior rehabilitation therapists and was grounded in the Job Demands-Resources (JD-R) theoretical framework. The four NASA-TLX items (mental demand, temporal demand, effort, and frustration) were selected a priori, in consultation with experienced physiotherapists and occupational therapists from the study setting, to represent cognitive, temporal and affective aspects of rehabilitation work. Physical demand was captured by Borg RPE(a widely used and validated measure of perceived exercise intensity that is commonly applied in clinical and rehabilitation programs), and performance was treated as a clinical outcome rather than a difficulty component. Equal weighting of NASA and Borg_norm was chosen to balance cognitive–affective and physical dimensions and to keep the index simple for clinical use, reflecting the conceptual framework that both dimensions are equally important contributors to perceived rehabilitation difficulty, consistent with the Job Demands-Resources (JD-R) framework [13–15] and with prior qualitative research describing rehabilitation complexity as shaped by multiple contextual and professional factors [42]. Future studies could examine whether differential weighting improves predictive validity.

### Psychometric evaluation of RIDI components

#### Internal consistency and dimensionality.

Cronbach’s alpha with 95% bootstrap confidence intervals (1,000 resamples) was calculated for the four NASA-TLX items and for the combined five-item set (four NASA items plus Borg_norm). Dimensionality was examined using exploratory factor analysis (principal component analysis), retaining factors with eigenvalues >1 and inspecting factor loadings and variance explained by the first factor.

#### Construct validity.

Pearson correlations were computed between RIDI, the NASA composite and Borg_norm to assess the contribution of each component to the index and to examine expected relationships among measures.

#### Intervention classification and statistical analysis.

After cleaning, 122 intervention categories were identified. For intervention-level analyses, only categories with ≥5 sessions were retained to ensure stable estimates; less frequent categories were summarised descriptively. For each retained intervention, the number of sessions, mean RIDI, standard deviation, range and 95% confidence interval were calculated.

Descriptive statistics (means, standard deviations, medians, interquartile ranges and ranges) were used to summarise NASA-TLX items, Borg RPE, RIDI, session duration and therapist experience. RIDI scores were compared across the 12 clinical areas using one-way analysis of variance (ANOVA) with eta-squared (η²) as the effect-size measure. Pearson correlation coefficients examined associations between RIDI and session duration, therapist experience, NASA-TLX items and Borg_norm as indicators of construct and convergent validity. Quantitative analyses were conducted in Python using standard data-analysis libraries.

### Qualitative component

#### Study design and participants.

A descriptive qualitative approach with thematic analysis was used to explore sources and management of rehabilitation difficulty and to help explain variation in quantitative RIDI scores. Purposive sampling recruited 20 therapists (11 physiotherapists, 9 occupational therapists) from 7 clinical areas represented in the quantitative study. Inclusion criteria were: licensed physiotherapist or occupational therapist, ≥ 2 years of clinical experience and willingness to participate in a 45–90-minute interview or focus group. Recruitment, facilitated by clinical managers, continued until no substantively new themes emerged.

#### Data collection.

Ten individual semi-structured interviews and one focus group with 10 therapists were conducted (45–90 minutes each). All sessions were interviewer-administered in Arabic, audio-recorded with consent, transcribed verbatim and translated into English, with spot-checking by bilingual researchers. Interview and focus group guides covered experiences of rehabilitation difficulty, patient- and context-related factors, organisational constraints, decision-making and perceived implications for practice. Guides were piloted with two therapists and refined; final versions are provided in S1A Appendix and S1B Appendix.

#### Data analysis and trustworthiness.

Thematic analysis followed Braun and Clarke’s six-phase approach (familiarisation, initial coding, theme development, review, definition and reporting). Two experienced qualitative researchers independently coded all transcripts, then resolved discrepancies through discussion. Inter-coder agreement was calculated on approximately 30% of transcripts using percent agreement and Cohen’s kappa. Data saturation was monitored by tracking the appearance of new themes across transcripts and was judged reached when no new substantive themes appeared in the final interviews.

Rigor was supported through multiple strategies: use of both interviews and a focus group (method triangulation), iterative team discussions and peer debriefing, maintenance of an audit trail of coding and analytic decisions, member checking of a summary of preliminary findings with a subset of participants (n = 5) and reflexive note-keeping by coders. The qualitative component was conducted and reported in line with the COREQ checklist.

### Mixed-methods integration

Quantitative and qualitative strands were analysed separately and then integrated using joint display analysis. Themes were tabulated by clinical area and by higher- versus lower-difficulty areas (based on RIDI distributions) to examine how qualitative patterns converged with or diverged from quantitative RIDI scores. Integrated interpretation considered consistencies and discrepancies between strands to refine understanding of rehabilitation difficulty and potential applications of RIDI.

### Ethical considerations

This study was conducted in accordance with the Declaration of Helsinki and approved by the Institutional Review Board of King Abdullah International Medical Research Center (Approval No. 00000135525). All participants provided written informed consent for quantitative data collection and qualitative participation. Participation was voluntary, with freedom to withdraw at any time. Data were de-identified after collection, and only aggregated results are reported to protect confidentiality.

## Results

### Quantitative results

#### Sample characteristics.

**Fig 2:**
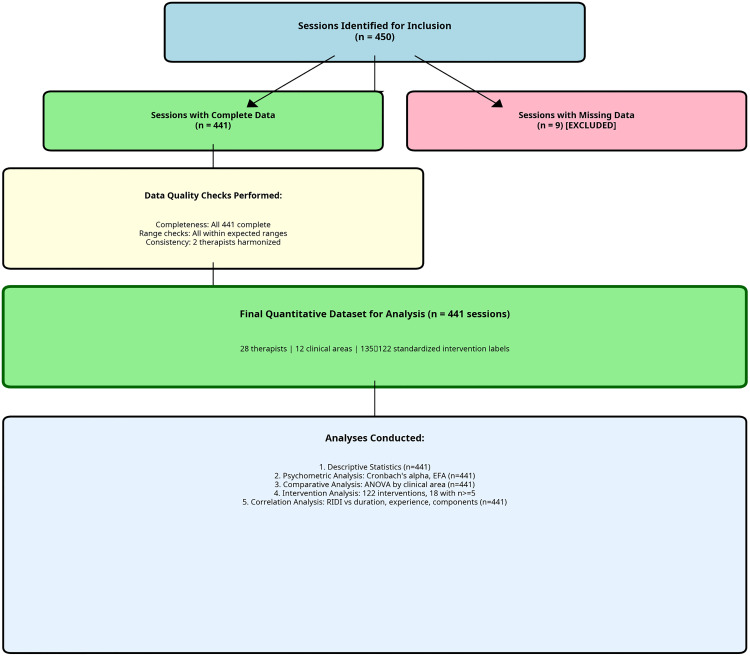
STROBE Flow Diagram for Quantitative Component. Flow diagram showing the selection and processing of rehabilitation sessions. Of 450 sessions identified for inclusion, 9 were excluded due to missing data. The remaining 441 sessions underwent data quality checks and intervention label standardization, resulting in the final dataset for analysis. All analyses conducted are listed at the bottom of the diagram.

The analysis dataset comprised 441 rehabilitation sessions conducted by 28 therapists across 12 clinical areas. The sample included sessions from acute neurorehabilitation, burn care, intensive care, internal medicine, orthopaedic rehabilitation, and outpatient services. Therapists had a mean of 7.5 years of experience (SD = 3.1, range 3–17 years). Session duration ranged from 1 to 120 minutes (M = 23.5, SD = 15.3). All 441 sessions had complete data for the key variables; no missing values were detected in the NASA-TLX items, Borg RPE, or session duration, the participant flow and data processing steps are presented in Fig 2.

#### RIDI scores and descriptive statistics.

RIDI scores were calculated for all sessions using the predefined formula. The NASA-TLX composite score was computed as the mean of four items (mental demand, temporal demand, effort, and frustration), each rated on a 0–10 scale. Borg RPE scores on the 6–20 scale were normalised to 0–10 using Borg_norm = (Borg_raw − 6)/ 14 × 10, and RIDI was defined as the mean of the NASA composite and Borg_norm, yielding a 0–10 scale. In the final dataset, RIDI scores ranged from 0.25 to 9.02 (M = 4.85, SD = 1.69).

**Fig 3:**
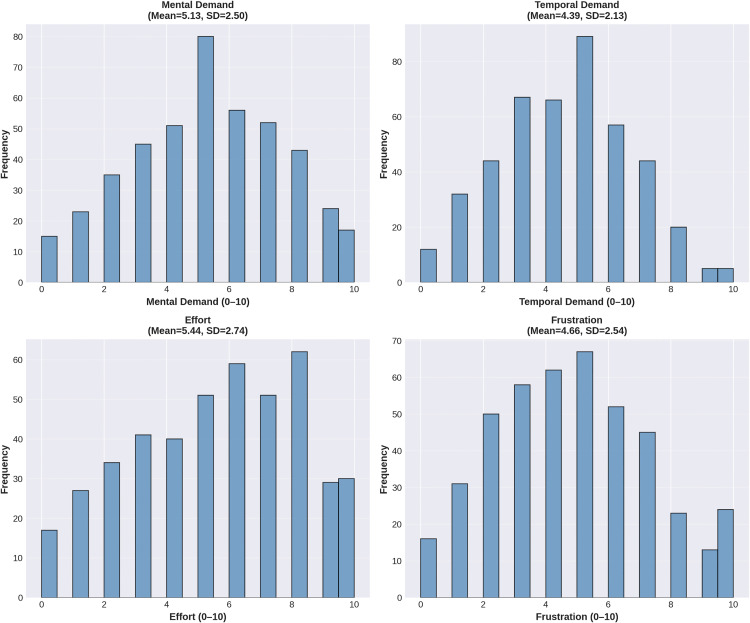
Distribution of NASA-TLX Dimensions Across All Rehabilitation Sessions. Histograms showing the frequency distribution of the four NASA-TLX workload dimensions (Mental Demand, Temporal Demand, Effort, Frustration) across 441 rehabilitation sessions, each rated on a 0–10 scale. All dimensions show approximately normal distributions with right-skew, indicating substantial variation in perceived difficulty across sessions. The distributions is consistent with the multidimensional approach to measuring rehabilitation difficulty.

Overall descriptive statistics are presented in Table 1. The NASA-TLX dimensions showed the following mean scores: mental demand (M = 5.13, SD = 2.50), temporal demand (M = 4.39, SD = 2.13), effort (M = 5.44, SD = 2.74), and frustration (M = 4.66, SD = 2.54). The NASA composite score had a mean of 4.91 (SD = 1.97). Borg RPE raw scores ranged from 6 to 20 (M = 12.71, SD = 3.26), with normalised scores ranging from 0 to 10 (M = 4.80, SD = 2.33). Fig 3 displays the mean scores and standard deviations for each NASA-TLX dimension.

**Table 1:** Overall Sample Descriptive Statistics.

Variable	n	Mean	SD	Median	IQR (25–75)	Min	Max
Mental Demand	441	5.13	2.50	5.00	3.00–7.00	0.00	10.00
Temporal Demand	441	4.39	2.13	4.00	3.00–6.00	0.00	10.00
Effort	441	5.44	2.74	6.00	3.00–8.00	0.00	10.00
Frustration	441	4.66	2.54	5.00	3.00–6.00	0.00	10.00
NASA_composite	441	4.91	1.97	5.00	3.50–6.50	0.25	9.25
Borg_raw	441	12.71	3.26	13.00	11.00–15.00	6.00	20.00
Borg_norm	441	4.80	2.33	5.00	3.57–6.43	0.00	10.00
RIDI	441	4.85	1.69	4.88	3.66–6.09	0.25	9.02
Session Duration (min)	441	23.45	15.32	20.00	12.00–30.00	1.00	120.00
Years of Experience	441	7.50	3.06	8.00	5.00–9.00	3.00	17.00

### RIDI by clinical area

**Fig 4:**
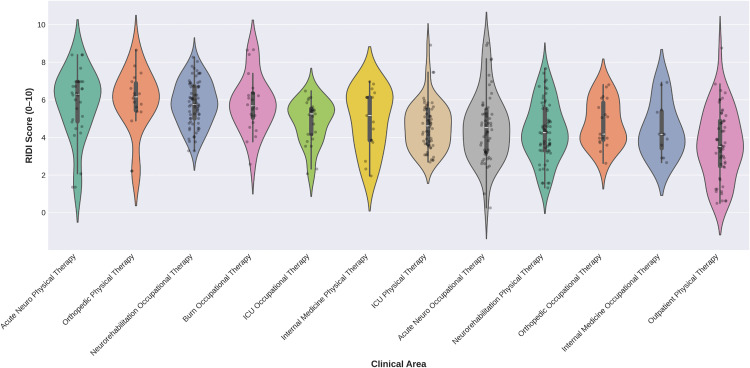
Distribution of Rehabilitation Intervention Difficulty Index (RIDI) Scores by Clinical Area. Violin plots showing RIDI score distributions across 12 clinical areas. Significant differences were found across areas (F(11,429) = 9.18, p < 0.001, η² = 0.19).

RIDI scores differed significantly across the 12 clinical areas (Table 2). A one-way analysis of variance (ANOVA) showed a statistically significant effect of clinical area on RIDI, F(11, 429) = 9.18, p < 0.001, with a medium effect size (η² = 0.19). The highest mean RIDI scores were observed in Neurorehabilitation Occupational Therapy (M = 5.80, SD = 1.20) and Acute Neuro Physical Therapy (M = 5.80, SD = 1.84), indicating that interventions in these specialties were perceived as more difficult. In contrast, Outpatient Physical Therapy had the lowest mean RIDI score (M = 3.63, SD = 1.87), suggesting that outpatient sessions were perceived as less demanding. Fig 4 illustrates the distribution of RIDI scores across clinical areas, showing medians, quartiles, and individual data points for each specialty.

**Table 2:** RIDI by Clinical Area.

Clinical Area	n	Mean RIDI	SD	Min	Max
Acute Neuro Occupational Therapy	54	4.53	1.82	0.25	9.02
Acute Neuro Physical Therapy	30	5.80	1.84	1.36	8.39
Burn Occupational Therapy	24	5.79	1.49	2.57	8.66
ICU Occupational Therapy	25	4.83	1.16	2.07	6.46
ICU Physical Therapy	56	4.61	1.27	2.70	8.91
Internal Medicine Occupational Therapy	12	4.42	1.44	2.66	6.93
Internal Medicine Physical Therapy	17	4.95	1.64	1.95	6.96
Neurorehabilitation Occupational Therapy	69	5.80	1.20	3.29	8.27
Neurorehabilitation Physical Therapy	63	4.32	1.56	1.32	7.66
Orthopedic Occupational Therapy	19	4.74	1.23	2.62	6.80
Orthopedic Physical Therapy	18	5.94	1.65	2.21	8.64
Outpatient Physical Therapy	54	3.63	1.87	0.50	8.75

### Preliminary psychometric properties

#### Internal consistency.

Cronbach’s alpha was calculated for the NASA-TLX items and for the five-item RIDI component set. The four NASA-TLX items demonstrated good internal consistency (α = 0.80, 95% CI [0.77, 0.83]). When the four NASA items and the normalised Borg score were combined, internal consistency remained acceptable (α = 0.75, 95% CI [0.72, 0.78]), indicating that the cognitive/affective (NASA) and physiological (Borg) components form a reasonably coherent set of indicators of perceived intervention difficulty in this sample.

#### Factor structure.

Exploratory factor analysis revealed a unidimensional structure underlying the five RIDI component items. One factor had an eigenvalue greater than 1 (eigenvalue = 2.61) and explained 52.1% of the total variance. All five items loaded meaningfully on this factor: mental demand (loading = 0.75), temporal demand (0.77), effort (0.83), frustration (0.80), and normalised Borg RPE (0.37). The lower loading for Borg RPE is expected, as it reflects a physiological measure distinct from the cognitive and affective NASA-TLX items, but it still contributes to the overall construct. These loadings, presented in S2 Table, provide preliminary support for a single underlying dimension of rehabilitation intervention difficulty.

### Intervention-level difficulty

After standardising intervention labels to correct spelling errors and merge near-duplicate terms, 135 raw labels were consolidated into 122 standardised intervention categories. To ensure stable estimates, only interventions with at least 5 sessions were included in the main analysis, yielding 18 intervention categories.

The top 10 interventions ranked by mean RIDI are shown in Table 3. Transfer training and splinting had the highest mean difficulty ratings (M = 6.78 and 6.71, respectively), whereas passive range of motion (PROM) had the lowest mean RIDI (M = 2.68). The complete list of all 18 interventions with n ≥ 5 sessions, including their sample sizes and variability measures, is provided in Supplementary S3 Table. Interventions with fewer than 5 sessions (n = 104 categories) were retained in the dataset for descriptive purposes but were not included in formal statistical comparisons to avoid unstable estimates.

**Table 3:** Top Interventions (n ≥ 5, ranked by Mean RIDI).

Intervention	n	Mean RIDI	SD	Min	Max
Transfer Training	6	6.78	0.94	5.96	8.39
Splinting	6	6.71	2.08	3.59	8.66
Sitting on EOB	10	6.08	0.82	4.75	7.43
Caregiver and patient education	6	5.75	0.97	4.57	6.96
Standing balance training	6	5.58	1.00	3.66	6.38
Intern Education And Guidance	6	5.23	1.60	3.29	7.39
Sit to stand	6	5.16	1.67	3.20	7.68
Gait training	9	5.12	1.99	2.32	8.39
Activities of daily living (ADL) training	12	5.11	1.65	2.66	9.02
Education and guidance	10	4.87	0.99	3.54	6.12

**Fig 5:**
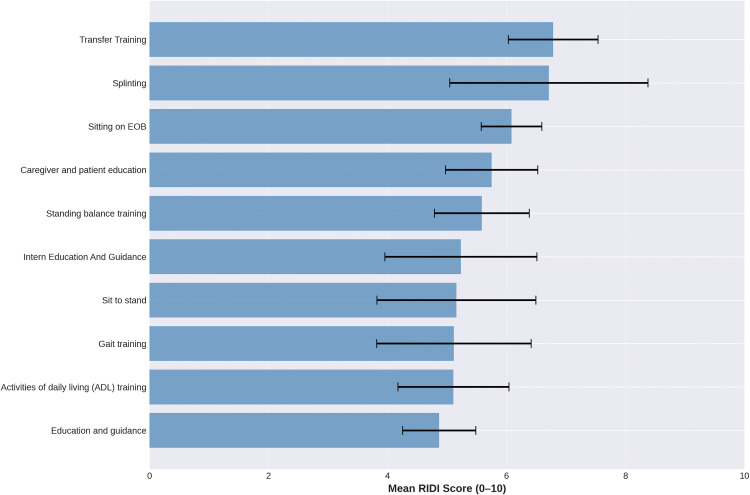
Mean Rehabilitation Intervention Difficulty Index (RIDI) Scores for Top 10 Interventions (n ≥ 5). Horizontal bar chart showing mean RIDI scores (0–10 scale) with 95% confidence intervals for the 10 most frequently performed interventions. Transfer Training and Splinting demonstrate the highest difficulty, while Education and guidance shows the lowest.

Fig 5 displays mean RIDI scores and 95% confidence intervals for the top 10 interventions, providing a visual comparison of the relative difficulty of commonly used interventions. Narrower intervals (e.g., for sitting on the edge of bed [EOB] with n = 10) indicate more precise estimates than wider intervals for interventions with smaller sample sizes.

### Correlation analyses

A Pearson correlation matrix for all key variables is presented in Table 4. The NASA-TLX items showed moderate to strong intercorrelations (r = 0.41–0.70, all p < 0.001), indicating that they measure related but distinct dimensions of perceived workload. The normalised Borg RPE showed weak to moderate correlations with the NASA items (r = 0.14–0.23, all p < 0.01), suggesting that the physiological exertion measure captures information that is partly independent from the cognitive and affective dimensions.

**Table 4:** Pearson Correlation Matrix.

Variable	Mental Demand	Temporal Demand	Effort	Frustration	NASA_composite	Borg_norm	RIDI	Duration	Experience
Mental Demand	1.000	0.586**	0.455**	0.407**	0.765**	0.142**	0.543**	0.237**	0.039
Temporal Demand		1.000	0.458**	0.419**	0.751**	0.226**	0.592**	0.331**	0.067
Effort			1.000	0.701**	0.843**	0.202**	0.629**	0.159**	0.067
Frustration				1.000	0.809**	0.183**	0.596**	0.055	0.037
NASA_composite					1.000	0.235**	0.743**	0.237**	0.066
Borg_norm						1.000	0.825**	0.205**	−0.036
RIDI							1.000	0.279**	0.013
Duration								1.000	0.055
Experience									1.000

Note: ** p < 0.01; * p < 0.05.

**Fig 6:**
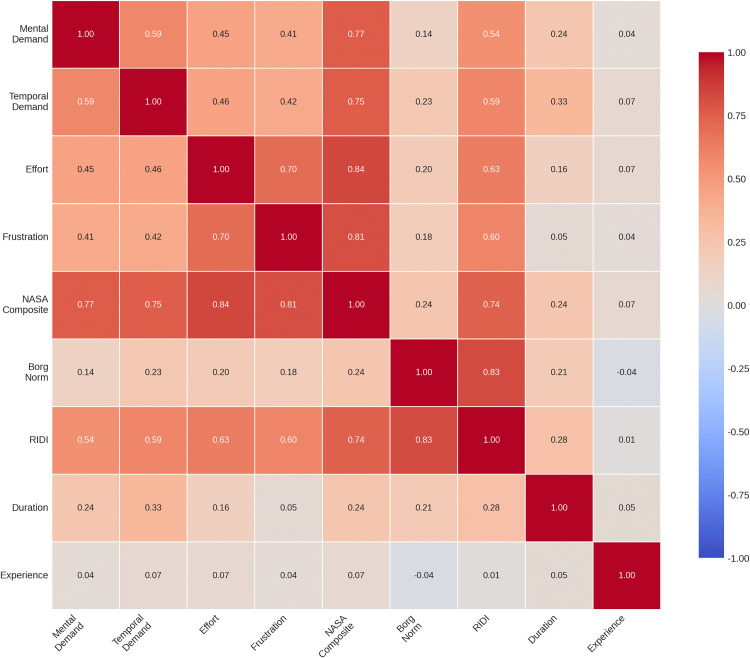
Pearson Correlation Matrix of RIDI Components and Related Variables. Heatmap showing pairwise correlations among NASA-TLX items (Mental Demand, Temporal Demand, Effort, Frustration), NASA composite score, normalized Borg RPE, RIDI, session duration, and therapist years of experience. Color intensity indicates correlation strength (red = positive, blue = negative). Strong correlations among NASA items (r = 0.41–0.84) support the composite measure, while RIDI shows moderate correlation with session duration (r = 0.28) and negligible correlation with therapist experience (r = 0.01).

RIDI was moderately correlated with the NASA composite (r = 0.74, p < 0.001) and strongly correlated with normalised Borg RPE (r = 0.83, p < 0.001), confirming that both components contribute substantially to the overall index. The correlation heatmap in Fig 6 provides a visual summary of all pairwise associations, with colour intensity representing correlation strength.

### Relationships with session duration and therapist experience

**Fig 7:**
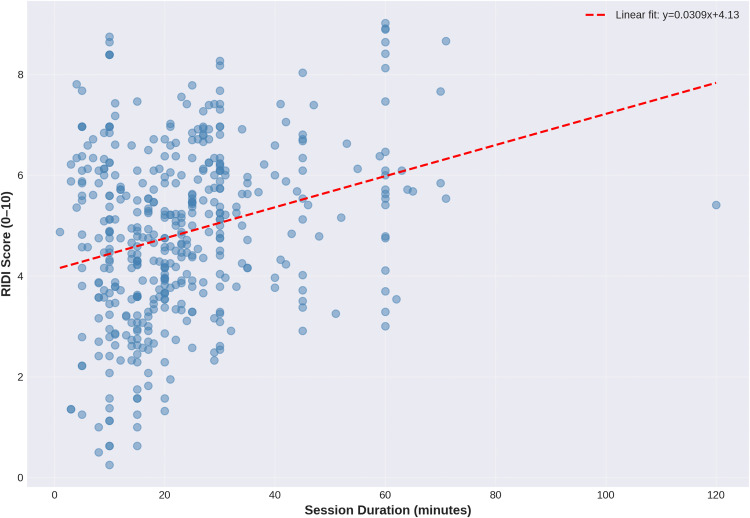
Relationship between RIDI Score and Session Duration. Scatterplot showing the positive association between session duration (minutes) and RIDI score (0–10 scale) across 441 rehabilitation sessions. The linear regression line (y = 0.0309x + 4.13) indicates that longer sessions are associated with higher rehabilitation difficulty (r = 0.28, p < 0.001).

**Fig 8:**
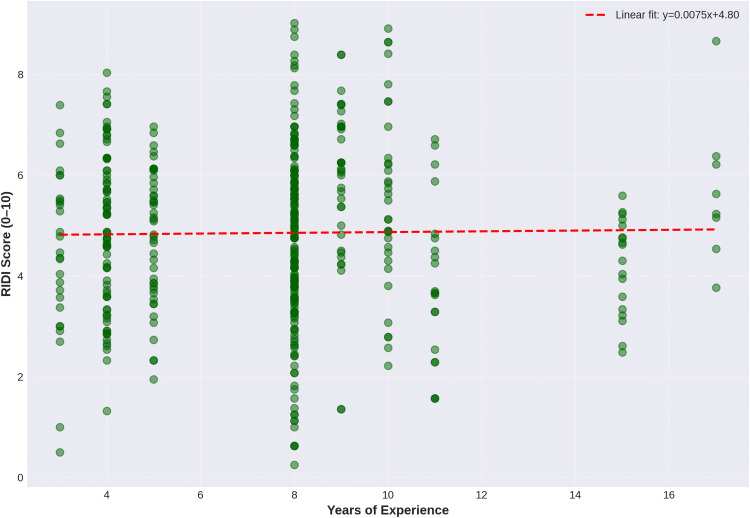
Relationship between RIDI Score and Therapist Years of Experience. Scatterplot showing no significant association between therapist years of experience and RIDI score across 441 rehabilitation sessions. The near-horizontal regression line (y = 0.0075x + 4.80) indicates that rehabilitation difficulty is independent of therapist experience (r = 0.01, p = 0.778).

As hypothesised, RIDI showed a moderate positive correlation with session duration (r = 0.28, p < 0.001; Fig 7), indicating that longer sessions tended to involve higher perceived intervention difficulty. Normalised Borg RPE also showed a weak positive correlation with duration (r = 0.21, p < 0.001), consistent with greater physiological exertion in longer treatments. In contrast, RIDI was not significantly correlated with therapist years of experience (r = 0.01, p = 0.78), suggesting that, within this setting, perceived intervention difficulty does not systematically vary by therapist seniority. Fig 8 (RIDI vs years of experience) illustrates this lack of association.

### Distribution of RIDI scores

**Fig 9:**
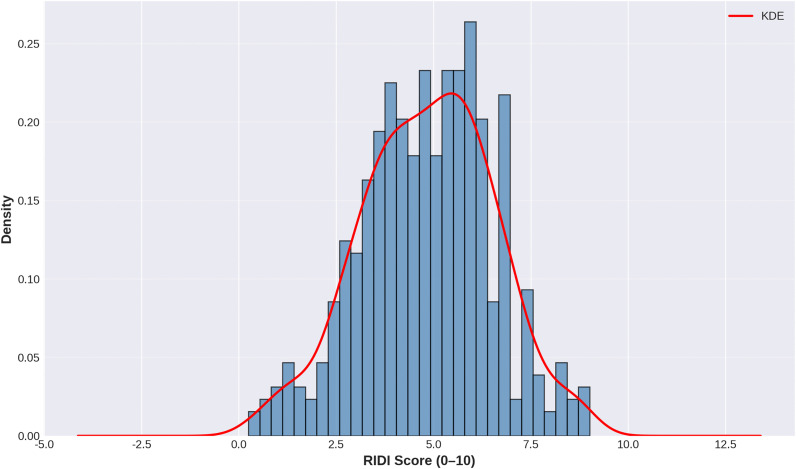
Distribution of RIDI Scores Across All Rehabilitation Sessions. Histogram with kernel density estimate (KDE) showing the distribution of RIDI scores (0–10 scale) across 441 rehabilitation sessions. The approximately normal distribution (M = 4.85, SD = 1.41) with slight right-skew indicates that most rehabilitation interventions cluster around moderate difficulty, with some sessions perceived as highly demanding.

The distribution of RIDI scores is shown in Fig 9 (RIDI distribution). RIDI values were approximately normally distributed with slight left skew, ranging from 0.25 to 9.02. The median RIDI score (4.88) was very close to the mean (4.85), supporting approximate normality. Individual NASA-TLX items also had a good spread across the 0–10 scale, with only small proportions of ratings at the floor (0) or ceiling (10), indicating no major floor or ceiling effects that would limit discriminative ability.

### Qualitative results

#### Overview.

Six main themes were identified from 349 coded segments contributed by all 20 participants (10 individual interviews, 10 focus group participants). The themes emerged through systematic coding of interview and focus group transcripts and were organised to reflect the multifaceted nature of rehabilitation difficulty. By approximately 70% of the coded dataset, no substantially new themes were identified, suggesting that thematic saturation had been reached. The qualitative findings provide rich contextual understanding of the quantitative RIDI patterns and illuminate the mechanisms through which rehabilitation interventions create demands on therapists.

### Theme-by-theme analysis

#### Theme 1: Time demands.

Time pressure and temporal constraints emerged as the most frequently discussed aspect of rehabilitation difficulty, with 78 coded segments from 18 participants. This theme encompassed discussions of session duration, scheduling pressures, the balance between clinical time and administrative tasks, and the challenge of completing complex interventions within limited timeframes. Participants consistently described time as a critical factor that shapes the overall difficulty of rehabilitation work. As one physiotherapist noted: “Documentation takes up so much time that could be spent with patients, and when you’re behind, the whole session feels rushed and harder.” The prominence of this theme across clinical areas suggests that temporal constraints are a near-universal experience in rehabilitation practice, although their manifestation differs by setting.

#### Theme 2: Cognitive demands.

Cognitive demands, including clinical reasoning, decision-making, and mental fatigue, were discussed in 72 coded segments from 18 participants. This theme captured the intellectual work of rehabilitation, such as assessing patient status, modifying interventions in real time, prioritising competing clinical needs, and managing complex patient presentations. Participants described the mental effort required to maintain focus and make sound clinical decisions, particularly in high-acuity settings. An occupational therapist working in neurorehabilitation reflected: “Neurological rehabilitation challenges you. You’re constantly prioritizing, analyzing, and making real-time decisions about what the patient can handle.” The high frequency of cognitive demand discussions, especially in acute care settings, underscores that rehabilitation difficulty is not merely physical but deeply cognitive.

#### Theme 3: Physical demands.

Physical demands, including patient handling, positioning, sustained physical effort, and postural strain, were identified in 65 coded segments from 17 participants. This theme encompassed the bodily exertion required to deliver rehabilitation interventions, from transferring patients to maintaining therapeutic positions during treatment. Participants discussed both acute physical effort (e.g., lifting a heavy patient) and cumulative strain from repetitive movements and sustained postures throughout the day. A physiotherapist in acute neuro care stated: “Transfer training is physically demanding—you’re using your body as a guide and bearing the patient’s weight while teaching them. By the end of the day, your back and shoulders are exhausted.” The substantial representation of this theme across all clinical areas highlights that physical exertion remains a core component of rehabilitation work.

#### Theme 4: Patient-related factors.

Patient-related factors, including patient motivation, cooperation, cognitive ability, and clinical complexity, were discussed in 57 coded segments from 16 participants. This theme reflected how patient characteristics shape the difficulty of delivering rehabilitation. Participants described how unmotivated, cognitively impaired, or medically unstable patients required greater effort and more frequent intervention modifications. One physiotherapist noted: “When a patient is confused or uncooperative, even a simple intervention becomes much harder. You have to constantly re-explain, adjust your approach, and manage their behavior while delivering therapy.” The consistent mention of patient factors across clinical areas indicates that difficulty is not solely determined by the intervention itself but by the interaction between the intervention and the patient’s capacity to engage.

#### Theme 5: Environmental constraints.

Environmental constraints, including equipment availability, workspace limitations, team support, and organisational resources, were identified in 44 coded segments from 15 participants. This theme captured how the physical and organisational environment either facilitates or hinders rehabilitation delivery. Participants discussed inadequate equipment, crowded treatment spaces, lack of team support, and organisational barriers that increased the difficulty of their work. An occupational therapist in an ICU setting explained: “When equipment isn’t available or the space is cramped, you have to improvise and work around limitations. That adds mental and physical effort to every intervention.” While less frequently discussed than time and cognitive demands, environmental constraints were consistently mentioned as a source of additional difficulty.

#### Theme 6: Coping strategies.

Coping strategies, including preparation, teamwork, time management, and contingency planning, were discussed in 33 coded segments from 14 participants. This theme captured the proactive and reactive approaches therapists use to manage difficulty and maintain effectiveness. Participants described strategies such as pre-planning sessions, collaborating with colleagues, delegating tasks, and developing backup plans for challenging situations. A physiotherapist reflected: “I prepare my sessions in advance, have my equipment ready, and work closely with the nursing team. Good teamwork and planning make even difficult sessions feel more manageable.” Notably, this theme was less frequently discussed overall and was much less prominent in higher-RIDI areas, suggesting that in the most demanding contexts therapists may have fewer opportunities to employ deliberate coping strategies.

### Mixed-methods integration

The qualitative themes align with and extend the quantitative RIDI findings in several ways. Cognitive demands were most frequently discussed in Acute Neuro (17 segments) and ICU (15 segments) settings, which are among the clinical areas with the highest quantitative RIDI scores (e.g., Acute Neuro PT: 5.80; ICU settings: 5.79) (S4 Table). This convergence suggests that the RIDI’s inclusion of NASA-TLX cognitive items (mental demand, temporal demand, effort, frustration) captures a substantial component of rehabilitation difficulty that therapists recognise and articulate in qualitative interviews.

Time demands were discussed extensively across all clinical areas but with particular emphasis in lower-RIDI settings (45 segments in lower-RIDI areas vs. 33 in higher-RIDI areas) (S5 Table). This pattern suggests that while time pressure is universal, it may be experienced differently: in lower-RIDI settings, time constraints may be the primary source of difficulty, whereas in higher-RIDI settings time pressure may compound already high cognitive and physical demands.

Coping strategies showed the greatest contrast between high- and low-RIDI contexts, with markedly fewer coded segments in higher-RIDI areas (8 vs. 23 in lower-RIDI areas, a reduction of approximately 65%) (S5 Table). This pattern may indicate a qualitative difference in how difficulty is managed: in lower-RIDI contexts, therapists appear more able to draw on deliberate coping strategies to organise their work, whereas in higher-RIDI areas (particularly acute neuro, burns, and orthopaedic care) the intensity and complexity of demands may limit opportunities to implement such strategies.

Overall, the joint alignment of qualitative themes with quantitative RIDI patterns provides preliminary support for the construct validity of RIDI as a measure of rehabilitation intervention difficulty. The qualitative data help to explain the mechanisms underlying the quantitative scores: time pressure, cognitive load, physical exertion, patient complexity, environmental barriers, and variable access to coping strategies all contribute to the overall difficulty that RIDI captures. Together, the quantitative and qualitative findings offer a more comprehensive picture of rehabilitation difficulty across clinical areas and intervention types.

## Discussion

### Summary of key findings

This mixed-methods study developed and preliminarily evaluated the Rehabilitation Intervention Difficulty Index (RIDI), a brief measure that integrates cognitive/affective workload (NASA-TLX) and perceived exertion (Borg RPE) to capture rehabilitation intervention difficulty. Across 441 sessions from 28 therapists in 12 clinical areas, RIDI demonstrated good internal consistency for NASA-TLX items (α = 0.80) and acceptable internal consistency for the full five-item scale (α = 0.75), a unidimensional factor structure, and meaningful variation across clinical areas. Qualitative interviews and focus groups with 20 therapists identified six themes underpinning difficulty: time demands, cognitive demands, physical demands, patient-related factors, environmental constraints, and coping strategies. Overall, the mixed-methods integration suggests that perceived difficulty reflects a combined cognitive, physical, and contextual burden that therapists experience during routine practice.

### Convergence between quantitative and qualitative findings

#### High RIDI scores in acute, neuro, burn, and orthopaedic contexts.

Neurorehabilitation and acute inpatient settings showed some of the highest RIDI scores, and therapists in these areas consistently described strong time pressure, substantial cognitive demands, and high physical effort. This pattern fits prior descriptions of hospital rehabilitation work as context-dependent and shaped by complex and demanding situations [42]. In acute neurological care, discharge pressure and limited time frames can require rapid clinical reasoning and decision-making when judging rehabilitation potential and tolerance [43]. In burn care, high perceived difficulty is plausible because rehabilitation commonly involves early and ongoing positioning, exercise, and splinting, and pain can substantially limit tolerance within multidisciplinary care [44]. For orthopaedic and other physically demanding inpatient interventions, therapists’ accounts of high physical effort align with evidence that awkward postures and treating heavier patients are common exposures, and that workload and working posture are linked to musculoskeletal pain among physiotherapists [17,18].

#### Contextual and organisational constraints as “difficulty amplifiers”.

Therapists also emphasised environmental and organisational constraints that amplified difficulty, including limited resources, unit logistics, and competing team demands. In ICU services, barriers such as staffing allocation, competing workload demands, limited treatment time, and limited availability of equipment/materials have been described as key constraints on rehabilitation delivery [5,45]. These constraints are consistent with the broader view that complexity in hospital-based physiotherapy is strongly shaped by context-related factors, especially when time and resources are limited [42]. Together, the quantitative–qualitative alignment supports the interpretation that RIDI reflects a combined cognitive, physical, and contextual burden in day-to-day care.

#### Therapist role and demographics.

Therapist years of experience did not significantly influence RIDI scores, but an important difference emerged by professional role. Occupational therapists reported higher RIDI scores than physiotherapists, suggesting that perceived difficulty varies by discipline rather than reflecting only individual experience. This is plausible because occupational and physical therapists may differ in job demands and health-related outcomes even within the same healthcare system [46]. In practice, OT work may involve complex functional and cognitive assessment, safety judgement, and activities-of-daily-living planning, whereas PT work may more often emphasise mobility and motor control. In ICU settings, occupational therapists have also described service constraints (e.g., staffing barriers and limited structured processes such as goal-setting meetings) that can increase planning and coordination burden [5]. Collectively, these points suggest that workforce planning should not assume uniform perceived difficulty across rehabilitation disciplines.

A limitation is that the quantitative dataset did not include therapist gender. Gender is commonly examined in occupational therapy burnout research and may relate to stress and burnout patterns, although findings can vary across samples [19]. Future RIDI studies should include gender and test whether associations with perceived difficulty and coping differ by setting and role.

#### Cognitive demands as a core component of difficulty.

Cognitive demands were especially prominent in Acute Neuro and ICU, where therapists described prioritising, analysing, and making real-time decisions about what patients could safely tolerate. This aligns with evidence from acute stroke and traumatic brain injury care indicating that discharge pressure can force assessment within limited time frames and require rapid clinical decision-making, with clinical reasoning described as a context-dependent process guiding action [43]. In ICU settings, feasibility pressures such as limited staffing, limited treatment time, and coordination barriers within the multidisciplinary team may further increase planning and decision-making burden [5,45]. This supports the inclusion of NASA-TLX in RIDI, as NASA-TLX is widely used to measure perceived cognitive workload and has demonstrated good test–retest reliability in applied measurement work [47]. Overall, these findings strengthen preliminary construct validity by indicating that perceived difficulty in hospital rehabilitation reflects both cognitive and physical demands shaped by clinical context [42].

#### Novel insights from mixed-methods integration: the coping strategies paradox.

A striking pattern was the under-representation of coping strategies in high-RIDI contexts compared with lower-RIDI contexts. In lower-RIDI settings, therapists described practical coping approaches such as prioritising tasks, time management, boundary setting, and seeking support, which are similar to coping strategies reported by occupational therapists facing ongoing work demands [48]. This also fits evidence that coping is partly shaped by job resources and organisational conditions, including autonomy and social support, which can buffer work pressure [9,48].

In contrast, therapists in high-RIDI contexts rarely mentioned coping strategies despite describing greater demands. This may indicate that the intensity and feasibility constraints of these settings leave limited opportunity to apply deliberate strategies during care delivery. This interpretation is consistent with ICU literature describing staffing/caseload limitations and restricted time for treatment, which can reduce real-time flexibility for planning and coordination [5,45]. In burn rehabilitation, where early rehabilitation is closely linked to pain control, positioning, and intensive management, the scope for reducing perceived difficulty through individual strategies alone may also be limited [44].

This finding suggests that high-RIDI areas may require structural and system-level responses rather than relying mainly on individual coping. Suggested approaches include staffing and caseload adjustments, improving equipment and space resources, and strengthening unit-level communication and coordination systems [45]. In parallel, occupational therapy evidence indicates that burnout is influenced by institutional support and resources, and that supportive management and workplace environments are linked to better resilience [19].

### Interpretation of RIDI patterns across clinical areas and interventions

#### Why outpatient settings have lower RIDI scores.

Outpatient physical therapy had the lowest RIDI score. Qualitative findings suggest this reflects a different pattern of difficulty rather than an absence of difficulty. In outpatient care, therapists often work with more medically stable patients and more predictable presentations, which may reduce constant risk management and rapid changes to treatment plans. This aligns with the view that rehabilitation complexity is shaped by context and patient-related factors, and that hospital-based work becomes more difficult when conditions change quickly and coordination demands increase [42]. Consistent with this, therapists described coping strategies as more feasible in outpatient settings. This fits evidence that perceived work pressure is closely related to control of time and job resources such as autonomy and social support [9], and that coping responses to work stress often involve time-management behaviours and seeking support [48]. In acute ICU contexts, structural constraints may limit the impact of coping strategies [5,45].

#### Intervention-level variation in RIDI.

Transfer training and splinting showed the highest RIDI scores, whereas PROM showed the lowest. Transfer training likely generates high perceived difficulty because it combines high physical effort, close manual handling, and continuous adaptation to patient tolerance and safety, often in medically complex patients. This aligns with evidence that patient transfer and maintaining/changing body position are key determinants of physiotherapy workload [3], and with evidence that awkward working positions and dealing with heavy patients are common job-related factors linked to work-related musculoskeletal problems in OT/PT [18]. Work posture and high workload have also been associated with musculoskeletal pain risk among physiotherapists [17]. Splinting may also be perceived as difficult because it involves decision-making and problem-solving within clinical constraints; in burn rehabilitation, splints are described as part of comprehensive rehabilitation to prevent/manage contractures and maintain anti-contracture positioning, especially when pain or limited compliance reduces tolerance of positioning alone [44]. Overall, these patterns reinforce that perceived difficulty reflects not only the intervention label but also the typical clinical context and patient profile in which the intervention is delivered [42].

#### Relationships with session duration and therapist experience.

RIDI showed a moderate positive correlation with session duration, suggesting longer sessions may be perceived as more difficult because they involve more complex content or sustained interaction. This is consistent with concerns that workload approaches based mainly on recorded time or activity volume can be limited and may not capture appropriateness without considering case mix and contextual factors [8]. It also aligns with rehabilitation ergonomics work indicating that workload can be driven by task demands and the wider work system rather than time alone [2]. The only moderate association reinforces that duration alone is an incomplete proxy for rehabilitation difficulty.

By contrast, RIDI showed no meaningful association with therapist years of experience. This suggests RIDI may reflect inherent session and context demands more than individual skill alone. Case-mix allocation may also contribute if experienced clinicians are more likely to manage complex patients or high-demand settings. This interpretation is consistent with evidence that perceived work pressure is strongly shaped by organisational factors such as time control and role conflict [9], and with findings that differences in reported job demands between OT and PT were not explained by demographics including seniority [46].

#### Psychometric properties and construct validity.

RIDI showed good internal consistency for NASA-TLX items and acceptable internal consistency for the five-item scale, with all components loading on a single factor. The lower loading of Borg RPE relative to NASA-TLX is consistent with its different focus, but still suggests perceived exertion contributes meaningfully to the overall construct. Conceptually, this fits qualitative work describing hospital rehabilitation complexity as reflecting an interplay between context/patient factors and therapist-related factors, drawing on cognitive and psychomotor competences during real-world decision-making [42]. RIDI correlations with the NASA composite and Borg_norm further support that the index captures a blended cognitive/affective and exertional experience of difficulty.

These findings provide preliminary support for the reliability and construct validity of RIDI in this single-centre sample. They are consistent with evidence that NASA-TLX can be a useful and psychometrically acceptable measure of perceived mental workload in applied assessments, supporting its inclusion within a composite difficulty index [47]. Nevertheless, more comprehensive validation is needed. Future studies should examine test–retest reliability, inter-rater reliability, and predictive validity in relation to outcomes such as burnout and work-related musculoskeletal problems, which have been linked to demanding work conditions, high workload, and job stress in rehabilitation professionals [17,18,48].

## Limitations

Several limitations should be noted. First, the study was conducted in a single tertiary hospital and included 441 sessions from 28 therapists; findings may not generalise to other institutions or models of care. Second, RIDI relies on self-reported perceptions of difficulty and may be influenced by response bias or local culture; objective workload measures were not collected. Third, the cross-sectional session-level design does not assess how difficulty accumulates over time or relates to longer-term outcomes such as retention or patient outcomes. Fourth, the qualitative sample included a subset of therapists and focused on seven clinical areas, and experiences in less-represented areas may not be fully captured. Fifth, many intervention categories were infrequently used, limiting stability of intervention-level estimates. Sixth, RIDI scores were self-reported by the assigned therapist, and inter-rater reliability was not assessed. While RIDI measures perceived difficulty (a subjective construct that may legitimately vary between therapists), future studies should examine whether different therapists would assign similar RIDI scores to the same intervention performed with the same patient, to establish the reliability of the measure across raters. Finally, psychometric evaluation was limited to internal consistency and exploratory factor analysis; larger studies should examine test–retest reliability and confirmatory factor analysis.

### Implications for clinical practice and research

#### Clinical practice.

RIDI may help teams map perceived difficulty across services and interventions, identify high-demand contexts, inform staffing and resource allocation, support workforce planning, and guide targeted service improvement efforts.

#### Research.

Priorities include inter-rater and test–retest reliability; predictive validity for workforce and clinical outcomes; validation across settings and cultures; deeper investigation of demographic and role-related differences; and triangulation with objective workload measures. Future work should also test whether system-level interventions (staffing, workflow redesign, environmental modification, clinical support) reduce RIDI and improve workforce outcomes.

## Conclusion

This mixed-methods study provides initial evidence that RIDI is a brief, feasible index capturing rehabilitation difficulty across clinical areas and interventions. Quantitative results showed acceptable reliability, a unidimensional structure, variation across settings, and plausible associations with session duration. Qualitative findings clarified key sources of difficulty and suggested that coping strategies are mentioned less often in high-demand contexts, highlighting the importance of organisational and structural support. Further validation across settings and longitudinal designs is required to establish predictive utility and to test whether RIDI-informed interventions can improve workforce wellbeing and patient care.

## Supporting information

S1 Table Data Validation and Cleaning. (DOCX)

S2 Table Psychometric Properties of RIDI Components. (DOCX)

S3 Table All Interventions. (DOCX)

S4 Table Joint Display by Clinical Area. (DOCX)

S5 Table Joint Display by RIDI Level. (DOCX)

S1A Appendix Individual Interview Guide. (DOCX)

S1B Appendix Focus Group Discussion Guide. (DOCX)
