# Supplementary material for: Developing a rehabilitation intervention difficulty index: A mixed-methods study using NASA-TLX and Borg RPE in a tertiary clinical setting
Source: PLoS One. 2026 Jan 12;21(1):e0340770. doi: 10.1371/journal.pone.0340770 (PMC12795390; doi:10.1371/journal.pone.0340770)
Supplement: S1 Table — (DOCX) [file pone.0340770.s001.docx]

Table S1: Data Validation and Cleaning

Data Confirmation

- Sessions: 441 (confirmed)
- Therapists: 28 (confirmed)
- Clinical Areas: 12 (confirmed)
- Intervention Labels: 135 raw labels (cleaned to 122 standardized categories)

Therapist-Level Summary

| Metric | Value |
| --- | --- |
| Mean sessions per therapist | 15.75 |
| SD | 5.58 |
| Min | 5 |
| Max | 23 |

Intervention Labels

| **Raw_Label** | **Cleaned_Label** |
| --- | --- |
| AAROM | Active-assisted range of motion (AAROM) |
| ADL Simulation (Dressing) | Adl Simulation (Dressing) |
| ADL training | Activities of daily living (ADL) training |
| ADL training (feeding) | Activities of daily living (ADL) training |
| ADLs | Activities of daily living (ADL) training |
| ADLs training | Activities of daily living (ADL) training |
| AFO application | Orthotic application |
| Active Transfer to chair | Active Transfer To Chair |
| Acute Medical Neuro | Acute Medical Neuro |
| Ambulation | Ambulation |
| Assistve Device Training | Assistve Device Training |
| Balance training | Balance training |
| Bed Mobility | Bed mobility |
| Bed mobility | Bed mobility |
| Bed mobility Training | Bed Mobility Training |
| Bed mobility training | Bed mobility |
| Bed positioning | Bed Positioning |
| Bed to Chair | Bed To Chair |
| Bedside ADL Training (Feeding) | Bedside Adl Training (Feeding) |
| Bilateral Arm Coordination | Bilateral Arm Coordination |
| Cargiver and paient education | Caregiver and patient education |
| Cognition training | Cognition Training |
| Cognitive Dual-Task Training | Cognitive Dual-Task Training |
| Contracture manegment | Contracture Manegment |
| Coordination Exercises | Coordination Exercises |
| Coordination exercises | Coordination training |
| Decumantation | Decumantation |
| Documentaion | Documentaion |
| Documentation | Documentation |
| Documintation | Documentation |
| Dry needle application | Dry Needle Application |
| Energy Conservation Education | Energy Conservation Education |
| Equipment Prescription | Equipment Prescription |
| Equipment prescription | Equipment Prescription |
| Equipment training | Equipment Training |
| Erego machine | Erego Machine |
| Family Education | Family Education |
| Fine motor exercises | Fine Motor Exercises |
| Functional Electrical Stimulation | Functional Electrical Stimulation |
| Functional Mobility (Bathroom) | Functional Mobility (Bathroom) |
| Functional ambulation | Functional Ambulation |
| Gait Training | Gait Training |
| Gait training | Gait training |
| Hand splinting | Hand Splinting |
| Hotpack application | Hotpack Application |
| ICU physical therapy team | Icu Physical Therapy Team |
| Ice application | Ice Application |
| Inhibition & Facilitation (NDT) | Inhibition & Facilitation (Ndt) |
| Intern Education | Intern Education |
| Intern Education and guidance | Education and guidance |
| Intern education and guidance | Intern Education And Guidance |
| Interns Education | Education and guidance |
| Interns education | Interns Education |
| Joint Mobilization | Joint Mobilization |
| Joints compression exercises | Joints Compression Exercises |
| Kitchen Task Simulation | Kitchen Task Simulation |
| March in place | March In Place |
| Memory Strategy Training | Memory Strategy Training |
| Mirror Therapy | Mirror Therapy |
| Mobility training | Mobility Training |
| Multidisciplinary team meeting | Multidisciplinary Team Meeting |
| Nurse education | Nurse Education |
| Obstacle Navigation Training | Obstacle Navigation Training |
| Orientation & Attention Drills | Orientation & Attention Drills |
| Orientation training | Orientation Training |
| PNF | Pnf |
| PROM | Passive range of motion (PROM) |
| Pasive T/F to chair | Pasive T/F To Chair |
| Passive Transfer | Passive Transfer |
| Patient & family education | Patient & Family Education |
| Patient File Screening | Patient File Screening |
| Positioning training | Positioning Training |
| ROM Exricise | Rom Exricise |
| ROM and stretching | Rom And Stretching |
| ROM ex's | Rom Ex'S |
| ROM exercises | Rom Exercises |
| ROM exes | Rom Exes |
| Scar management | Scar Management |
| Sensory Re-education | Sensory Re-Education |
| Shockwave | Shockwave |
| Sit to Stand | Sit to stand |
| Sit to stand training | Sit To Stand Training |
| Sitting Balance training | Sitting Balance Training |
| Sitting EOB | Sitting on EOB |
| Sitting EOB / Balance training | Sitting Eob / Balance Training |
| Sitting balance ex | Sitting Balance Ex |
| Sitting on EOB | Sitting on EOB |
| Sitting training | Sitting training |
| Soft Splinting | Soft Splinting |
| Splint fabrication | Splint Fabrication |
| Splinting | Splinting |
| Stair Training | Stair Training |
| Standing | Standing |
| Standing Balance Tranining | Standing balance training |
| Strengethening exes | Strengethening Exes |
| Strenghnining Training | Strengthening training |
| Strengthening exercises | Strengthening Exercises |
| Strengthing Exrcise | Strengthing Exrcise |
| Stretching Exrcise | Stretching Exrcise |
| T/F to tilting table | T/F To Tilting Table |
| Tactile Discrimination Training | Tactile Discrimination Training |
| Taping | Taping |
| Toilet Transfer Practice | Toilet Transfer Practice |
| Traction application | Traction Application |
| Tranfer training | Tranfer Training |
| Transfer Training | Transfer Training |
| Transfering Training | Transfering Training |
| Transfers training | Transfers Training |
| Trigger Points release | Trigger Points Release |
| Upper Limb Task Practice | Upper Limb Task Practice |
| Visual Scanning Training | Visual Scanning Training |
| Visual-Motor Coordination Games | Visual-Motor Coordination Games |
| Wheelchair Propulsion Trng | Wheelchair Propulsion Trng |
| Wound care | Wound Care |
| Writing/Graphomotor Re-training | Writing/Graphomotor Re-Training |
| ambulating complex case like ECMO or pt on mechanical vent | Ambulating Complex Case Like Ecmo Or Pt On Mechanical Vent |
| balance training | Balance Training |
| celling hoist | Celling Hoist |
| cycling | Cycling |
| documentation | Documentation |
| electrotherapy | Electrotherapy |
| family education | Family Education |
| hot pack | Hot Pack |
| intern education & guidance | Intern Education & Guidance |
| intern education and guidance | Intern Education And Guidance |
| marching in place | Marching In Place |
| motomed | Motomed |
| scapula moblization | Scapula Moblization |
| spine brace application | Spine Brace Application |
| stair climbing | Stair Climbing |
| stairs training | Stairs Training |
| stretching | Stretching |
| tiliting table | Tiliting Table |
| transfer training | Transfer Training |
| treadmill | Treadmill |

Data Quality

- Missing values: None detected in key variables
- Out-of-range values: None detected
- Data inconsistencies identified: 2 therapists (Q_025, Q_027) with multiple "Years of Experience" values
  - Resolution: Used most frequent value for each therapist (Q_025: 3 years; Q_027: 8 years)

Documentation: See data_cleaning_log.txt
