# Supplementary material for: Developing a rehabilitation intervention difficulty index: A mixed-methods study using NASA-TLX and Borg RPE in a tertiary clinical setting
Source: PLoS One. 2026 Jan 12;21(1):e0340770. doi: 10.1371/journal.pone.0340770 (PMC12795390; doi:10.1371/journal.pone.0340770)
Supplement: S2 Table — (DOCX) [file pone.0340770.s002.docx]

**Table S2:** Psychometric Properties of RIDI Components

| **Measure** | **Value** | **95% CI Lower** | **95% CI Upper** |
| --- | --- | --- | --- |
| NASA-TLX (4 items) | 0.8013 | 0.7675 | 0.8275 |
| RIDI components (5 items) | 0.7542 | 0.7178 | 0.7836 |
| First factor eigenvalue | 2.6094 | — | — |
| Variance explained (%) | 52.07 | — | — |
