# Supplementary material for: Developing a rehabilitation intervention difficulty index: A mixed-methods study using NASA-TLX and Borg RPE in a tertiary clinical setting
Source: PLoS One. 2026 Jan 12;21(1):e0340770. doi: 10.1371/journal.pone.0340770 (PMC12795390; doi:10.1371/journal.pone.0340770)
Supplement: S3 Table — (DOCX) [file pone.0340770.s003.docx]

Table S3. All Interventions

| **Intervention** | **n** | **Mean RIDI** | **SD** | **Min** | **Max** |
| --- | --- | --- | --- | --- | --- |
| Transfer Training | 6 | 6.78 | 0.94 | 5.96 | 8.39 |
| Splinting | 6 | 6.71 | 2.08 | 3.59 | 8.66 |
| Sitting on EOB | 10 | 6.08 | 0.82 | 4.75 | 7.43 |
| Caregiver and patient education | 6 | 5.75 | 0.97 | 4.57 | 6.96 |
| Standing balance training | 6 | 5.58 | 1.00 | 3.66 | 6.38 |
| Intern Education and Guidance | 6 | 5.23 | 1.60 | 3.29 | 7.39 |
| Sit to stand | 6 | 5.16 | 1.67 | 3.20 | 7.68 |
| Gait training | 9 | 5.12 | 1.99 | 2.32 | 8.39 |
| Activities of daily living (ADL) training | 12 | 5.11 | 1.65 | 2.66 | 9.02 |
| Education and guidance | 10 | 4.87 | 0.99 | 3.54 | 6.12 |
| Family Education | 5 | 4.84 | 1.11 | 3.91 | 6.71 |
| Documentation | 19 | 4.46 | 1.47 | 2.07 | 7.05 |
| Strengthening training | 6 | 4.42 | 1.63 | 3.07 | 6.71 |
| Bed mobility | 12 | 4.38 | 1.09 | 2.62 | 6.96 |
| Sitting training | 6 | 4.36 | 1.50 | 2.91 | 6.96 |
| Equipment Prescription | 5 | 3.42 | 1.99 | 0.25 | 5.59 |
| Active-assisted range of motion (AAROM) | 6 | 3.35 | 1.53 | 1.36 | 5.59 |
| Passive range of motion (PROM) | 6 | 2.68 | 1.11 | 1.36 | 4.29 |
