# Supplementary material for: Developing a rehabilitation intervention difficulty index: A mixed-methods study using NASA-TLX and Borg RPE in a tertiary clinical setting
Source: PLoS One. 2026 Jan 12;21(1):e0340770. doi: 10.1371/journal.pone.0340770 (PMC12795390; doi:10.1371/journal.pone.0340770)
Supplement: S4 Table — (DOCX) [file pone.0340770.s004.docx]

**Table S4: Joint Display by Clinical Area**

| **Setting** | **Theme** | **n_seg** | **n_part** |
| --- | --- | --- | --- |
| Acute Neuro | Time demands | 14 | 4 |
| Acute Neuro | Cognitive demands | 17 | 4 |
| Acute Neuro | Physical demands | 13 | 4 |
| Acute Neuro | Patient-related factors | 13 | 4 |
| Acute Neuro | Environmental constraints | 10 | 4 |
| Acute Neuro | Coping strategies | 3 | 3 |
| Burn | Time demands | 5 | 2 |
| Burn | Cognitive demands | 6 | 2 |
| Burn | Physical demands | 5 | 2 |
| Burn | Patient-related factors | 6 | 2 |
| Burn | Environmental constraints | 2 | 1 |
| Burn | Coping strategies | 2 | 1 |
| ICU | Time demands | 13 | 4 |
| ICU | Cognitive demands | 15 | 4 |
| ICU | Physical demands | 22 | 4 |
| ICU | Patient-related factors | 13 | 3 |
| ICU | Environmental constraints | 9 | 3 |
| ICU | Coping strategies | 11 | 4 |
| Internal Medicine | Time demands | 11 | 3 |
| Internal Medicine | Cognitive demands | 4 | 3 |
| Internal Medicine | Physical demands | 5 | 2 |
| Internal Medicine | Patient-related factors | 9 | 3 |
| Internal Medicine | Environmental constraints | 8 | 3 |
| Internal Medicine | Coping strategies | 5 | 2 |
| Neurorehabilitation | Time demands | 10 | 3 |
| Neurorehabilitation | Cognitive demands | 13 | 3 |
| Neurorehabilitation | Physical demands | 7 | 3 |
| Neurorehabilitation | Patient-related factors | 5 | 2 |
| Neurorehabilitation | Environmental constraints | 7 | 2 |
| Neurorehabilitation | Coping strategies | 4 | 2 |
| Orthopedic | Time demands | 14 | 2 |
| Orthopedic | Cognitive demands | 6 | 2 |
| Orthopedic | Physical demands | 4 | 2 |
| Orthopedic | Patient-related factors | 5 | 2 |
| Orthopedic | Environmental constraints | 7 | 2 |
| Orthopedic | Coping strategies | 3 | 2 |
| Outpatient | Time demands | 11 | 2 |
| Outpatient | Cognitive demands | 11 | 2 |
| Outpatient | Physical demands | 9 | 2 |
| Outpatient | Patient-related factors | 7 | 2 |
| Outpatient | Environmental constraints | 2 | 2 |
| Outpatient | Coping strategies | 3 | 2 |
