# Supplementary material for: Developing a rehabilitation intervention difficulty index: A mixed-methods study using NASA-TLX and Borg RPE in a tertiary clinical setting
Source: PLoS One. 2026 Jan 12;21(1):e0340770. doi: 10.1371/journal.pone.0340770 (PMC12795390; doi:10.1371/journal.pone.0340770)
Supplement: S5 Table — (DOCX) [file pone.0340770.s005.docx]

**Table S5: Joint Display by RIDI Level**

| Theme | Higher RIDI (n_seg) | Lower RIDI (n_seg) | Ratio |
| --- | --- | --- | --- |
| Time demands | 33 | 45 | 0.73 |
| Cognitive demands | 29 | 43 | 0.67 |
| Physical demands | 22 | 43 | 0.51 |
| Patient-related factors | 24 | 34 | 0.71 |
| Environmental constraints | 19 | 26 | 0.73 |
| Coping strategies | 8 | 23 | 0.35 |
