# Supplementary material for: Developing a rehabilitation intervention difficulty index: A mixed-methods study using NASA-TLX and Borg RPE in a tertiary clinical setting
Source: PLoS One. 2026 Jan 12;21(1):e0340770. doi: 10.1371/journal.pone.0340770 (PMC12795390; doi:10.1371/journal.pone.0340770)
Supplement: S1A Appendix — (DOCX) [file pone.0340770.s006.docx]

Appendix A: INDIVIDUAL INTERVIEW GUIDE (English Version)

Study: Developing a Rehabilitation Intervention Difficulty Index: A Mixed-Methods Study Using NASA-TLX and Borg RPE in a Tertiary Clinical Setting

Duration: 60-90 minutes

Format: Semi-structured individual interview

Recording: Audio recorded with participant consent

---

INTERVIEW PROTOCOL

OPENING (5-10 minutes)

Rapport Building and Context Setting

1. Background and Experience

- Can you tell me about your role here and how long you've been working in rehabilitation?

- What clinical areas do you primarily work in?

- How would you describe a typical day in your work?

2. Warm-up Question

- When you think about your work as a therapist, what makes some days feel more challenging than others?

---

MAIN INTERVIEW TOPICS

A. INTERVENTION COMPLEXITY AND DIFFICULTY (15-20 minutes)

3. Defining Difficulty

- How would you define what makes a rehabilitation intervention "difficult" or "complex"?

- Can you give me an example of an intervention you recently found particularly challenging?

- Probe: What specifically made it challenging?

- Probe: How did you handle those challenges?

4. Variation in Complexity

- Do you notice differences in how difficult interventions are across different clinical areas?

- Probe: Which areas tend to be most/least demanding? Why?

- How does intervention difficulty vary between different types of patients?

- Probe: What patient characteristics make interventions more complex?

5. Intervention-Specific Factors

- Are there specific types of interventions that you consistently find more demanding?

- Probe: What about activities like transfers, gait training, ADL training?

- Probe: What makes these interventions particularly challenging?

B. PATIENT-RELATED COMPLEXITY FACTORS (10-15 minutes)

6. Patient Characteristics

- How do patient factors influence the difficulty of your interventions?

- Probe: Medical complexity, comorbidities?

- Probe: Cognitive status, communication abilities?

- Probe: Motivation and psychological factors?

7. Challenging Patient Scenarios

- Can you describe a patient case where multiple factors made interventions particularly complex?

- How do you adapt your approach when working with patients who have communication barriers?

- Probe: Language barriers, aphasia, cognitive impairment?

C. ENVIRONMENTAL AND ORGANIZATIONAL FACTORS (10-15 minutes)

8. Work Environment

- How does your physical work environment affect the difficulty of interventions?

- Probe: Space constraints, equipment availability?

- Probe: Noise, interruptions, crowding?

9. Organizational Pressures

- How do time pressures and scheduling demands affect your work?

- Tell me about how workload and patient assignments impact intervention complexity.

- Probe: Too many patients, not enough time?

- Probe: Documentation requirements?

10. Resource Availability

- How does equipment availability or sharing affect your interventions?

- What happens when you don't have the ideal resources for a treatment?

D. PROFESSIONAL DECISION-MAKING AND CLINICAL REASONING (10-15 minutes)

11. Clinical Decision-Making

- Walk me through how you assess and plan for a complex intervention.

- How do you make real-time adjustments during treatment sessions?

- Probe: What factors do you consider?

- Probe: How do you balance competing demands?

12. Interdisciplinary Collaboration

- How does working with other disciplines affect intervention complexity?

- Probe: Coordinating care, conflicting recommendations?

- Probe: Communication challenges?

E. WORKLOAD ASSESSMENT AND MEASUREMENT (10-15 minutes)

13. Current Workload Assessment

- How do you currently think about or measure your workload?

- Do you feel that traditional measures (like number of patients) capture the reality of your work?

- Probe: What's missing from current approaches?

14. Mental vs. Physical Demands

- How would you compare the mental versus physical demands of your work?

- Are there interventions that are physically demanding but mentally easy, or vice versa?

15. Experience and Expertise

- How has your perception of intervention difficulty changed as you've gained experience?

- Do you think experienced and novice therapists would rate the same intervention differently?

F. IMPLICATIONS AND APPLICATIONS (5-10 minutes)

16. Workload Management

- If you could design the ideal way to assess and manage workload in rehabilitation, what would it look like?

- How could better workload assessment help with staffing and resource allocation?

17. Training and Support

- What kind of support or training would help therapists manage complex interventions better?

---

CLOSING (5 minutes)

18. Additional Thoughts

- Is there anything important about intervention complexity that we haven't discussed?

- What advice would you give to someone trying to understand the demands of rehabilitation work?

19. Study Feedback

- Do you have any questions about this research?

- Would you be interested in hearing about the results when the study is complete?

---

INTERVIEWER GUIDELINES

Before the Interview:

- Review participant's demographic information

- Ensure recording equipment is working

- Prepare comfortable, private setting

- Have consent forms ready

During the Interview:

- Begin with rapport building

- Use open-ended questions

- Allow natural flow while covering all topics

- Use probes to encourage detailed responses

- Take notes on non-verbal cues and context

- Maintain neutral, non-judgmental stance

Probing Techniques:

- Elaboration: "Can you tell me more about that?"

- Clarification: "What do you mean by...?"

- Examples: "Can you give me a specific example?"

- Feelings: "How did that make you feel?"

- Process: "Walk me through what happened..."

After the Interview:

- Complete field notes immediately

- Note any technical issues with recording

- Record initial impressions and themes

- Thank participant and provide contact information

---

SAMPLE PROBING QUESTIONS BY THEME

Patient Complexity:

- "You mentioned comorbidities - can you give me an example of how that affected your treatment?"

- "What does it look like when a patient has multiple complex conditions?"

- "How do you handle patients who are unmotivated or fearful?"

Environmental Factors:

- "Describe a time when space constraints made your job harder."

- "What happens when you're interrupted during a treatment session?"

- "How do you adapt when equipment isn't available?"

Clinical Reasoning:

- "Take me through your thought process during that intervention."

- "What were you weighing in your mind as you made that decision?"

- "How did you know to change your approach?"

Workload Assessment:

- "What would capture the 'real' difficulty of your work?"

- "How would you explain your workload to someone who's never done this job?"

- "What's missing from how workload is currently measured?"
