# Supplementary material for: Developing a rehabilitation intervention difficulty index: A mixed-methods study using NASA-TLX and Borg RPE in a tertiary clinical setting
Source: PLoS One. 2026 Jan 12;21(1):e0340770. doi: 10.1371/journal.pone.0340770 (PMC12795390; doi:10.1371/journal.pone.0340770)
Supplement: S1B Appendix — (DOCX) [file pone.0340770.s007.docx]

Appendix B: FOCUS GROUP DISCUSSION GUIDE (English Version)

Study: Developing a Rehabilitation Intervention Difficulty Index: A Mixed-Methods Study Using NASA-TLX and Borg RPE in a Tertiary Clinical Setting

Duration: 120 minutes

Participants: 10 therapists (diverse clinical areas and experience levels)

Facilitator: External researcher (not from rehabilitation department)

Recording: Audio recorded with participant consent

---

FOCUS GROUP PROTOCOL

OPENING (10 minutes)

1. Welcome and Introductions

- Welcome and thank participants

- Brief study overview and purpose

- Ground rules for discussion

- Confidentiality assurance

- Audio recording consent confirmation

2. Participant Introductions

- "Please introduce yourself with your first name, your role, and how many years you've been in rehabilitation."

3. Ice Breaker

- "Let's start by going around the room. Think of one word that describes your work as a rehabilitation therapist."

- Facilitator records words on flip chart

---

MAIN DISCUSSION TOPICS

A. DEFINING INTERVENTION COMPLEXITY (20 minutes)

4. Group Definition Building

- "As a group, how would you define what makes a rehabilitation intervention complex or difficult?"

- Facilitator creates list on flip chart as participants contribute

- Probe: "Are there different types of complexity?"

- Probe: "What else should be on this list?"

5. Consensus and Disagreement

- "Looking at this list, which factors do you all agree are most important?"

- "Are there any factors that some of you see differently?"

- Facilitator marks areas of consensus and disagreement

6. Prioritization

- "If you had to choose the top 3 factors that make interventions complex, what would they be?"

- Allow discussion and gentle debate

B. CLINICAL AREA DIFFERENCES (25 minutes)

7. Area Comparisons

- "Let's talk about differences between clinical areas. Which areas do you find most demanding?"

- Probe: "Why do you think these differences exist?"

- Probe: "What makes ICU different from outpatient care?"

- Probe: "How does neurorehabilitation compare to orthopedics?"

8. Intervention Types Discussion

- "What specific interventions consistently challenge you the most?"

- Facilitator lists interventions mentioned

- "Let's try to rank these from most to least demanding. Do you all agree?"

- Allow discussion of disagreements

9. Patient Population Effects

- "How do different patient populations affect intervention complexity?"

- Probe: "Neurological vs. orthopedic vs. burn patients?"

- Probe: "Acute vs. chronic conditions?"

- "Do you all see similar patterns, or are there differences in your experiences?"

C. ENVIRONMENTAL AND SYSTEM FACTORS (20 minutes)

10. Workplace Environment

- "What environmental factors make your work more difficult?"

- Probe: "Space, equipment, noise, interruptions?"

- "How do these factors affect different clinical areas differently?"

11. Organizational Challenges

- "How do organizational factors affect intervention complexity?"

- Probe: "Staffing levels, time pressure, documentation requirements?"

- "Do you all experience these pressures similarly, or are there differences?"

12. Resource and Equipment Issues

- "Tell me about how equipment availability affects your work."

- "What happens when resources are limited or shared between units?"

- Allow participants to build on each other's experiences

D. WORKLOAD ASSESSMENT DISCUSSION (25 minutes)

13. Current Measurement Critique

- "How is your workload currently measured or assessed in your department?"

- "Does this capture the reality of your work? Why or why not?"

- Probe: "What's missing from current approaches?"

14. Ideal Assessment Tool

- "If you could design the perfect workload assessment tool, what would it include?"

- Facilitator captures ideas on flip chart

- "What would make it practical to use in daily practice?"

15. Mental vs. Physical Demands

- "How do you balance mental and physical demands in your work?"

- "Should these be measured separately or together? What do you think?"

- "Are there interventions that are high in one but low in the other?"

16. Experience and Perception

- "Do you think experience changes how we perceive intervention difficulty?"

- "Would a new graduate rate interventions the same way you do?"

- Allow experienced and newer therapists to share perspectives

E. TOOL VALIDATION AND FEEDBACK (15 minutes)

17. RIDI Concept Introduction

- Facilitator presents basic RIDI concept: combining mental workload (NASA-TLX) and physical effort (Borg RPE)

- "What do you think about this approach of combining mental workload and physical effort measures?"

- "Would this be useful in your practice?"

18. Implementation Considerations

- "What would make a workload assessment tool practical to use?"

- "What barriers might prevent its use in your daily work?"

- "How could this information be used to improve your work environment?"

19. Refinement Suggestions

- "What would you add or change about this approach?"

- "Are there important aspects of your work that this might miss?"

---

CLOSING (5 minutes)

20. Key Messages

- "What's the most important thing for administrators and managers to understand about your workload?"

- "If you could send one message about intervention complexity to hospital leadership, what would it be?"

21. Final Thoughts

- "Any final thoughts on intervention complexity or workload assessment?"

- "Is there anything important we haven't discussed?"

22. Next Steps

- Thank participants for their time and insights

- Explain how results will be shared with participants

- Provide contact information for follow-up questions

- Remind about confidentiality

---

FACILITATOR GUIDELINES

Pre-Focus Group Preparation:

- Review participant demographics for balanced representation

- Prepare flip chart paper and markers

- Test recording equipment

- Arrange seating in circle or U-shape

- Prepare name tags or tent cards

Facilitation Techniques:

Encouraging Participation:

- "What do others think about that?"

- "Does anyone have a different experience?"

- "[Name], you've been quiet - what's your perspective?"

- "Let's hear from someone who works in a different area."

Managing Dominant Speakers:

- "That's a great point, [Name]. Let's hear what others think."

- "I want to make sure we hear from everyone."

- Use non-verbal cues (eye contact with others)

Probing for Depth:

- "Can you give us a specific example?"

- "What does that look like in practice?"

- "Help us understand what you mean by..."

- "How do others handle that situation?"

Building on Ideas:

- "That builds on what [Name] said earlier..."

- "Are there other examples of this?"

- "Does this connect to what we discussed about...?"

Managing Group Dynamics:

Encouraging Disagreement:

- "It sounds like there might be different perspectives on this."

- "That's interesting - does everyone agree with that?"

- "What might explain these different experiences?"

Keeping on Track:

- "That's a great discussion. Let's make sure we also cover..."

- "I want to come back to that, but first let's finish talking about..."

- "We have about 10 minutes left for this topic."

Handling Sensitive Topics:

- Acknowledge emotions without taking sides

- Redirect to group experience rather than individual complaints

- Focus on systems rather than individuals

Documentation During Session:

- Key themes and quotes on flip chart

- Note areas of consensus and disagreement

- Record non-verbal reactions

- Track participation levels

- Note any technical issues

Post-Focus Group Tasks:

- Complete detailed field notes immediately

- Review recording quality

- Note initial themes and impressions

- Plan follow-up if needed

- Send thank you message to participants

---

SAMPLE DISCUSSION STARTERS

For Quiet Participants:

- "I'd like to hear from those who haven't spoken yet."

- "[Name], does this match your experience in [clinical area]?"

- "What would you add to what's been said?"

For Building Consensus:

- "It sounds like most of you agree that..."

- "Are there common themes emerging here?"

- "What seems to be the shared experience?"

For Exploring Differences:

- "I'm hearing different perspectives on this."

- "What might explain these different experiences?"

- "Are there factors that make this vary between therapists?"

For Practical Applications:

- "How would this work in practice?"

- "What would this look like on a typical day?"

- "How could this information be used?"
